# Comparison of Performance of Predicting the Wear Amount of Tire Tread Depending on Sensing Information

**DOI:** 10.3390/s23010459

**Published:** 2023-01-01

**Authors:** Kangjun Kim, Hyunjae Park, Taewung Kim

**Affiliations:** Department of Mechanical Design Engineering, Tech University of Korea, Siheung-si 15073, Republic of Korea

**Keywords:** tire wear prediction, machine-learning, tire internal acceleration, vehicle speed, tire internal pressure, tire vertical load

## Abstract

Excessive tire wear can affect vehicle driving safety. While there are various methods for predicting the tire wear amount in real-time, it is unclear which method is the most effective in terms of the difficulty of sensing and prediction accuracy. The current study aims to develop prediction algorithms of tire wear and compare their performances. A finite element tire model was developed and validated against experimental data. Parametric tire rolling simulations were conducted using various driving and tire wear conditions to obtain tire internal accelerations. Machine-learning-based algorithms for tire wear prediction utilizing various sensing options were developed, and their performances were compared. A wheel translational and rotational speed-based (V and ω) method resulted in an average prediction error of 1.2 mm. Utilizing the internal pressure and vertical load of the tire with the V and ω improved the prediction accuracy to 0.34 mm. Acceleration-based methods resulted in an average prediction error of 0.6 mm. An algorithm using both the vehicle and tire information showed the best performance with a prediction error of 0.21 mm. When accounting for sensing cost, the V and ω-based method seems to be promising option. This finding needs to be experimentally verified.

## 1. Introduction

Tire wear is one of the major factors affecting the driving safety of a vehicle. Excessive tire wear will have a big impact on the directional stability and braking performance of a vehicle in slippery road conditions [1]. Tires with a tread depth less than 1.6 mm, which is the guideline for a tire replacement, have been shown to have three times more likelihood of being involved in tire-related vehicular crashes than tires with tread depth ranging between 2.4 mm and 3.2 mm [2]. Therefore, regular monitoring of the tire wear state is necessary. Vehicle maintenance is also one of the key tasks in car-sharing businesses [3]. It is expected that automated monitoring of the tire condition will contribute to making the maintenance process more efficient. In that process, one of items to monitor will be the tread depth of the tire.

Although there have been many studies that have developed methods for estimating the amount of tire wear in real time, there has been a paucity of information comparing the performances of the various sensing options in estimating tire wear amount (see Section 2). Therefore, it is unclear which method is the most effective in terms of technological and economical standpoints because different studies have used different sensor information and driving conditions. The goal of this study is to develop an algorithm to predict the amount of tire wear by using vehicle and tire sensing information, and to quantitatively compare the performances of the algorithms based on various combinations of sensor data. First, a finite element model of a rubber-tired metro was developed and validated against static and dynamic test data. Second, a series of tire rolling simulations was performed under various wear amounts and driving conditions to obtain tire internal acceleration signals. Third, an algorithm for estimating tire wear in real time were developed by analyzing the extracted internal acceleration signals and driving information using various artificial intelligence techniques. Lastly, the performances of the various sensing options were quantitively compared.

## 2. Related Materials

Zhang et al. developed a model for estimating tire wear amount in real time by training a back-propagation neural network (BPNN) using a 3-axis accelerometer and strain gauge data attached to the inner surface of the tire [4]. A series of tire rolling tests was performed on tires with a wear rate between 0% and 75%, and an artificial intelligence algorithm was developed to predict wear by extracting features from the measured acceleration and strain data. The trained BPNN demonstrated that the prediction errors were within 0.05 mm. However, this method requires the installation of a 3-axis accelerometer and a strain gauge inside each tire. It also requires a networking device and data processing units. Similarly, Kim et al. conducted a driving test using three kinds of wear amounts (0%, 40%, 80%) while measuring the internal acceleration of the tire [5]. Then, a tire wear prediction algorithm was developed by using the frequency components of the radial acceleration signals. However, the algorithm was developed as a classifier rather than a regressor. Therefore, this method cannot predict values between 0%, 40%, and 80%. Li et al. used a finite element (FE) tire model to elucidate the relationships among the amount of tire wear, tire loading conditions, and frequency responses of the tire [6]. The authors showed that the frequency component of the radial acceleration changes according to the amount of tire wear, internal pressure, load, and speed of the running tire. Based on these observations, the authors developed an artificial neural network-based tire wear prediction algorithm using the above factors as features. The developed algorithm demonstrated an excellent prediction performance with an average wear rate prediction error of 2.8%. The proposed method is currently in the conceptual stage, and there is a need for a method for measuring the radial resonant frequencies of a rolling tire on a vehicle. Lastly, Poloni and Lu showed that the effective rolling radius can be predicted based on the wheel rotational and travel speeds [7]. The authors only demonstrated their method for a new tire, which has a single tread depth condition. Andrews et al. showed that the thickness of the tire tread can be measured based on the change of the electromagnetic field [8]. When an oscillating electrical signal was applied between the electrodes of the printed sensor using a metallic carbon nanotube ink, the capacitance and overlying material thickness showed a linear correlation. Therefore, this method eliminates the need for complex signal processing, which is necessary for smart sensing methods utilizing the accelerometer or strain gauge. However, this method requires the use of a special treatment inside the tire to allow for the capacitance change to be measured.

As was done by Li et al., the finite element method (FEM) has been widely used in research and development in the field of tire mechanics to reduce the time and cost involved in the experimental process, and its accuracy has been verified through many studies [9,10]. For the development of an intelligent tire system, it is very useful to investigate the feasibility of the considered concepts via computational approaches prior to prototype production. This is particularly true when prototyping or testing is not easy to perform due to the difficulty of measuring or performance limits of the equipment. Rosu et al. used the finite element tire model to predict the temperature change inside the tire until the time of takeoff of the airplane tire [11]. Behroozinia et al. proposed the possibility of detecting cracks inside the tire through analysis of the acceleration signal measured inside the tire using rolling simulations of the finite element tire model [12].

## 3. Methods

### 3.1. Development of Tire Model

#### Tire Modeling

**Figure 1:**
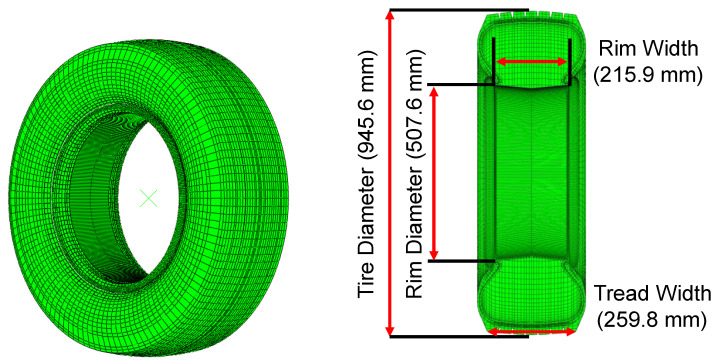
Dimension of the simple tread tire model.

A heavy-duty tire (315/70R20) used on the Busan Subway Line 4 was constructed as a finite element (FE) model using ABAQUS (Abaqus 6.21-1, Simulia, Dassault Systèmes, Paris, France). To create the model, we first developed a two-dimensional axisymmetric model based on a two-dimensional computer-aided design (CAD) drawing. This model consisted of 274 nodes and 345 elements and was made of 16 synthetic rubber materials and seven cord materials. Their Young’s moduli and specific gravities were determined using ASTM D412 and ASTM D297 for the rubber, and ASTM D2969 for the cord by the manufacture of the tire [13,14,15]. The Young’s moduli of the rubber materials ranged from around 2.5 MPa to 10 MPa, while those of the cord materials ranged from 110 GPa to 150 GPa. The specific gravity of the rubber materials had an average value of 1.1 gmm^−2^, while that of the cord materials ranged from 3.5 to 10.0 gmm^−2^. The Poisson ratios for the rubbers and the cords were set to 0.49 and 0.3, respectively. Then, a three-dimensional model was created by revolving it with 3-degree increments along the rotation axis of the tire, thus resulting in 120 sections (Figure 1). The outer diameter (OD) and width of the tire without internal pressure were 945.6 mm and 259.8 mm, respectively. The OD and width of the rim were 507.6 mm and 215.9 mm, respectively. Only the groove of the tread was considered without considering a detailed pattern of the tire.

### 3.2. Tire Model Validation

The developed tire model was validated using experimental data in three steps. First, the vertical and lateral stiffness of the tire model was compared with the quasi-static test data. Second, modal analysis results of the tire model were compared with the modal test data under three internal pressure conditions. Third, the normal component of the tire internal acceleration signals during two drum rolling simulations were compared with the experimental data.

#### 3.2.1. Validation of Rigidity

**Figure 2:**
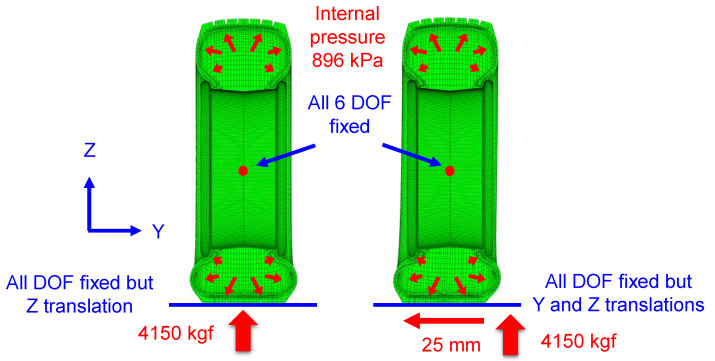
Boundary conditions for calculations of vertical and lateral stiffness of the tire model.

To verify the rigidity of the tire model, we compared the vertical and lateral stiffnesses of the model with those of the physical tire (Figure 2). The measurement errors of the test equipment for tire stiffness were less than 0.6% and 0.5% for forces and displacements, respectively. The stiffness of the tire model was calculated by performing a quasi-static simulation using the ABAQUS/Standard solver following the same procedures used for the physical tire. First, the vertical stiffness of the tire was calculated by applying an internal pressure of 896 kPa and a vertical load of 4150 kgf. Second, the floor surface was moved 11 mm in the pure lateral direction while maintaining the same vertical load and internal pressure. The force measured at the wheel center and the lateral displacement were used to calculate the lateral stiffness. Then, the stiffnesses of the tire model were compared with those of the physical tire in two modes: vertical and lateral.

#### 3.2.2. Validation of Modal Characteristics

**Figure 3:**
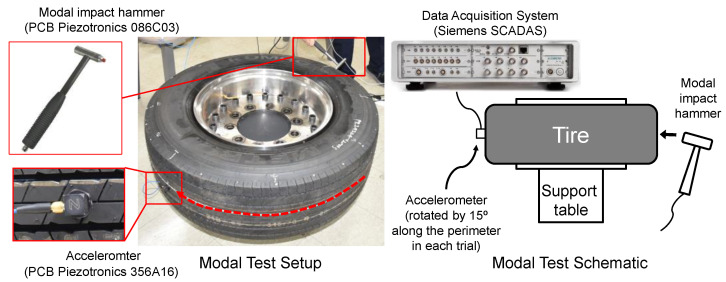
Modal test of physical tire using accelerometer and modal impact hammer.

The natural frequencies and mode shapes of the tire model were compared with those of the physical tire to verify the dynamic characteristics of the tire model. A set of modal tests was performed by applying internal pressures of 672 (85%), 896 (100%), and 1030 (115%) kPa to the physical tire [16]. It is important to note that the rim of the tire was set on the table without any load applied to the tire other than gravity (Figure 3). Three-axis accelerometers were positioned along the center line of the tire tread at intervals of approximately 15 degrees. Then, accelerations were measured with a sampling rate of 10 kHz while an impact hammer struck the tire. Modal analysis of the tire model was performed using the *FREQUENCY keyword provided by the ABAQUS/Standard solver for the above three internal pressure conditions. The modal frequencies and shapes of the physical tire were compared with those of the tire model.

#### 3.2.3. Validation of Internal Acceleration

**Figure 4:**
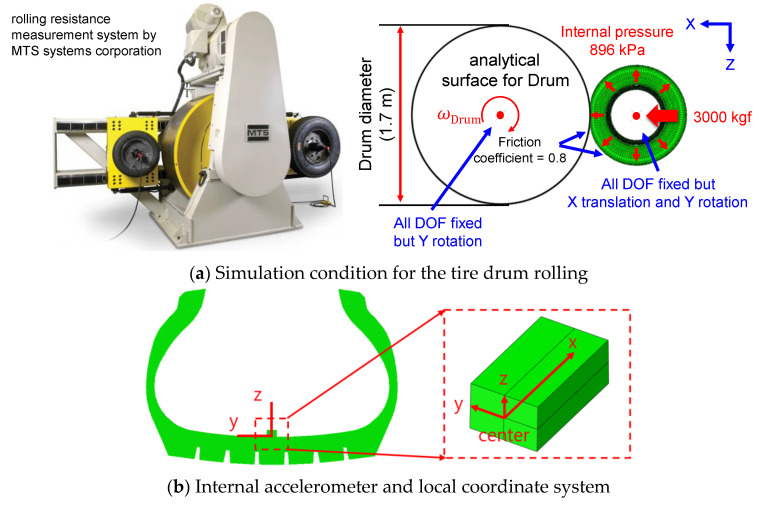
Comparison of tire internal acceleration under drum rolling conditions.

The tire model was validated under drum rolling conditions by comparing internal 3-axis acceleration signals against matched test data (Figure 4a). The drum rolling tests were performed using a tire rolling resistance measurement system for commercial truck tires manufactured by MTS Systems Corporation (Eden Prairie, MN, USA). The accuracies of the velocity of the drum surface and vertical load were within ±0.5 kmh^−1^ and ±10 N, respectively. The diameter of the drum in the test equipment was 1.7 m. During the drum rolling test, the acceleration signals were measured at a sampling rate of 10 kHz on the inner surface of the tire. The drum rolling tests were performed at two speeds: 40 km/h and 60 km/h. The vertical load and internal pressure applied to the tire were 4150 kgf and 896 kPa, respectively. Accelerometer elements were added to the tire model at the point where the accelerometer was installed during the test. A local coordinate system for the accelerometer model was constructed using the three nodes of the accelerometer element (Figure 4b). Then, the radial acceleration (z-axis) of the accelerometer was measured from the drum rolling simulations. The high-frequency components of the acceleration signals from the test and the simulation were filtered out using the CFC60 filter, a low-pass filter with a cut-off frequency of approximately 100 Hz [17]. Then, the radial accelerations from the test and the simulation were compared.

### 3.3. Generation of Virtual Tire Rolling Data

#### 3.3.1. Parametric Tire Rolling Simulation

**Figure 5:**
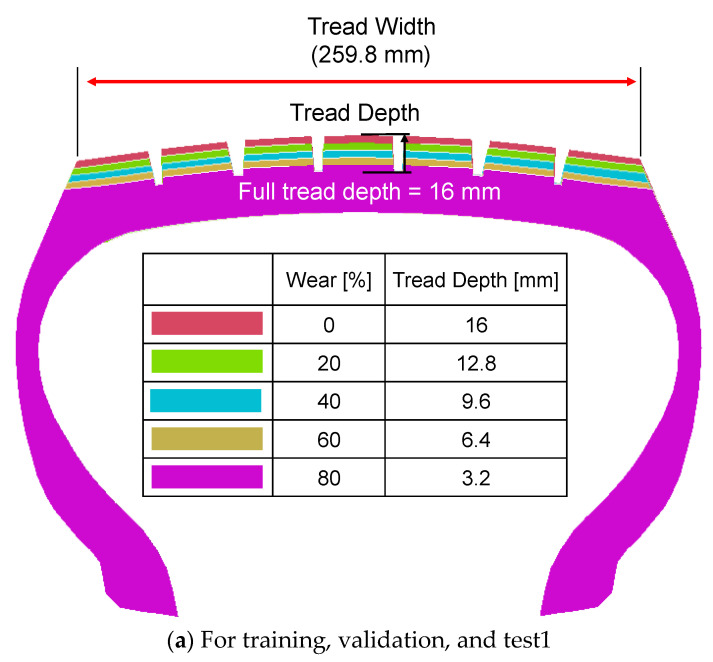

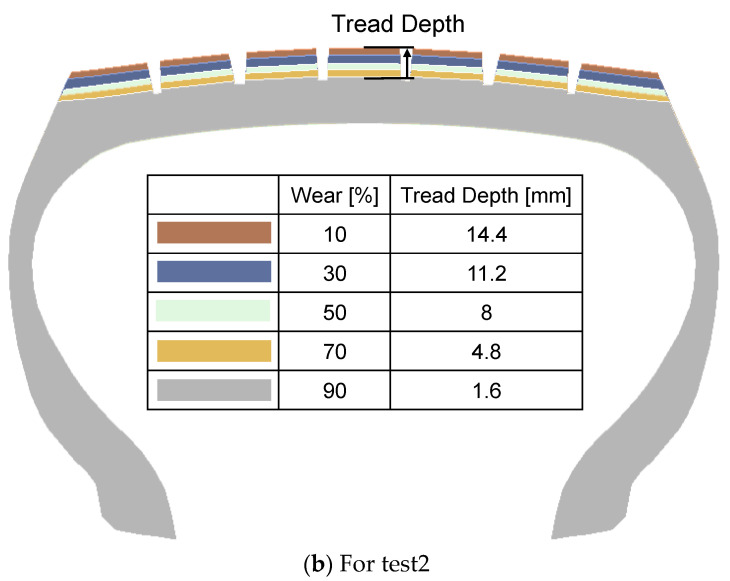
Set of tread depth conditions for rolling simulations.

Two sets of parametric straight-line driving simulations were performed on flat ground using the validated tire model to generate data for training and testing the tire wear prediction algorithm (Table 1). The parameters considered in the first set of parametric simulations included five tread depths, three vertical loads, three internal pressures, and four travel speeds. The initial tread depth of the tire model was 16 mm, which means 0% tread wear. The five tread wear conditions were 0%, 20%, 40%, 60%, and 80% (Figure 5a). These parameters resulted in 180 simulation conditions, which were simulated using the ABAQUS/Explicit solver. Another round of parametric tire rolling simulations was performed using the same methodology as the first series but with different sets of parameter values (Table 1). Instead of the first parametric simulations, five different wear amounts—10, 30, 50, 70, and 90%—were considered (Figure 5b). The first parametric simulation results were used for training, validation, and testing algorithms for tire wear prediction following the typical supervised learning process. Then, the second parametric simulation results were used to verify the generalization performance of the developed tire wear prediction algorithms (Table 1).

#### 3.3.2. Processing of Acceleration Signal

**Figure 6:**
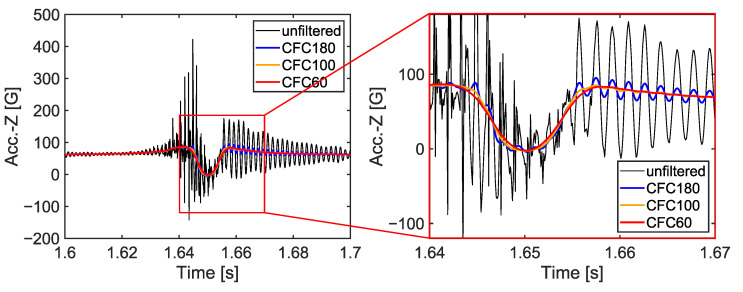
Average acceleration time histories from the three consecutive cycles.

The time histories of the tire internal acceleration and travel velocity were collected for each simulation. The collected tire internal accelerations, which were in the global coordinate system, were transformed into the accelerometer local coordinate system. As it has been done for the drum simulation results, the high-frequency components of the acceleration time histories were removed using the CFC60 filter [17] (Figure 6).

### 3.4. Development of Tire Wear Prediction Algorithm

#### 3.4.1. List of Features for Machine Learning

**Figure 7:**
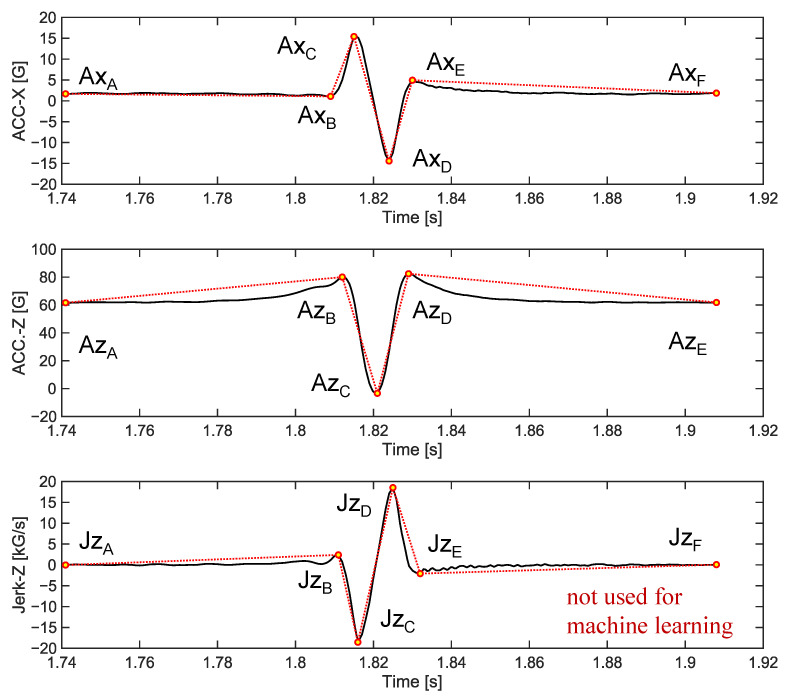
Points that were manually chosen to capture the characteristics of the acceleration signals.

**Figure 8:**
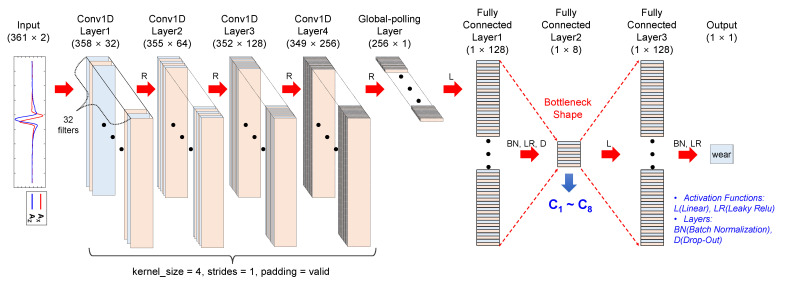
1D-CNN to extract eight bottleneck features for predicting the amount of tire wear.

**Figure 9:**
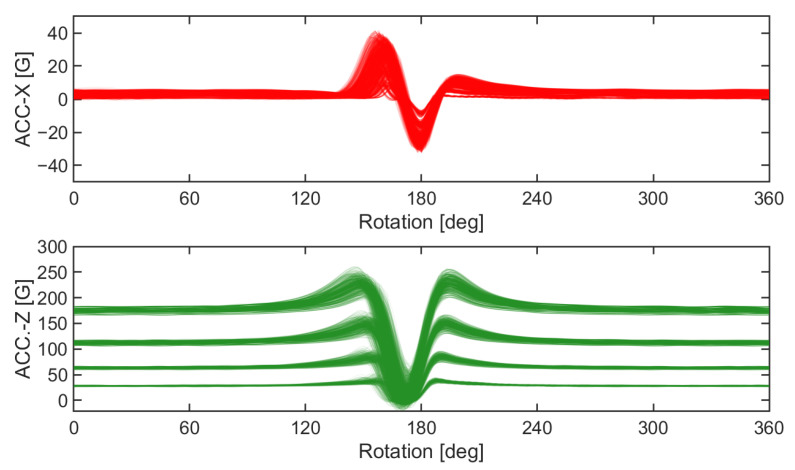
Time histories of circumferential and radial accelerations from tire rolling simulations on the flat road.

The vehicle conditions and tire conditions were used as features to develop ML-based wear prediction algorithms (Table 2). It was assumed that the information to be obtained from the vehicle was the travel speed of the tire (V), the rotational speed of the wheel (ω), and the vertical load (L) applied to the tire. The information obtained from the tire included the tire internal pressure (P) and the circumferential and radial components of the tire internal acceleration (a_x_ and a_z_, respectively). Initially, the time derivative of the a_z_, which is called a jerk, was also considered to be used as a feature, but the jerk signal was not used because of its similarity with the a_x_ (Figure 7). The features of the a_x_ and a_z_ time histories were chosen based on two methods. First, characteristic points of the tire internal accelerations were manually selected as features for ML by examining the shape of the a_x_ and a_z_ (Figure 7). Second, eight additional features (C_1_~C_8_) that appeared to be effective in predicting tire wear were extracted using 1D-CNN with a bottleneck structure (Figure 8). Since the dimensions of acceleration time series data per cycle differed depending on the wheel rotational speed, the dimension of each cycle of the acceleration signals was matched by converting the time axis into the rotation angle (Figure 9). The time axis was divided by 360 over the period of the current cycle so that cycle of the acceleration and jerk signals consisted of 361 data points, which represent one data point for 1 degree rotation. Then, the eight acceleration-related features were extracted using the 1D-CNN method (Figure 8). In this process, the 361-dimensional acceleration data was reduced to 160 dimensions using the four convolutional 1D layers. Then, a BPNN layer, which was designed to predict the wear, was used to predict the tire wear amount. One layer with eight nodes was placed in the middle of the BPNN so that the eight features were derived by training this hybrid network to predict the tire wear [18]. The effectiveness of the eight features for the 1D-CNN was investigated by cross-plotting each pair of the eight features with the tire wear information.

#### 3.4.2. Estimation of Tire Wear Using Machine Learning

**Figure 10:**
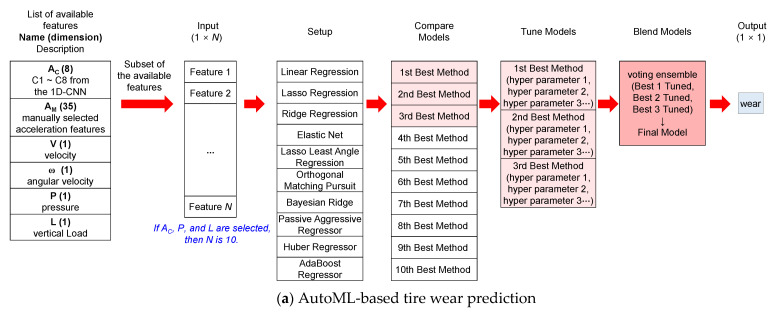

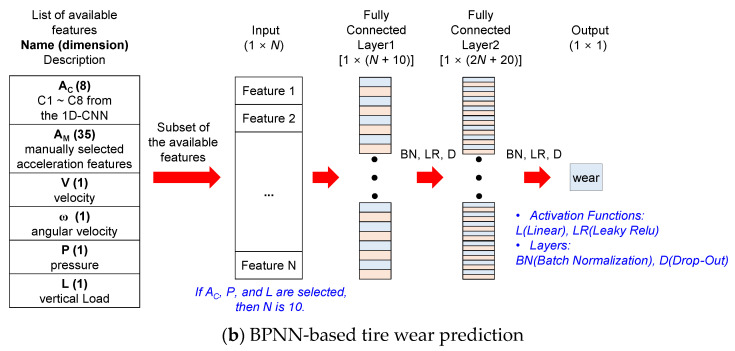
Schematic of the tire wear prediction.

The algorithms used to estimate tire wear amount were developed using the BPNN and the Automated Machine Learning (AutoML) method provided by PyCaret (version 2.3.10) [19]. First, the tire wear amount was predicted based on the features listed in Table 2 using AutoML, which is an ensemble method based on ten widely used ML algorithms, which include Linear Regression [20], Lasso Regression [21], Ridge Regression [22], Elastic Net Regression [23], Lasso Least Angle [24], Orthogonal Matching Pursuit [25], Bayesian Ridge [26], Passive Aggressive Regressor, Huber Regressor [27], and AdaBoost Regressor [28]. Most of the parameters for the ten ML methods provided by the AutoML function were kept as their default values (see Appendix A). Table 3 shows the list of parameters that were altered from their default values in the current study. The AutoML function provided by PyCaret (version 2.3.10) train the ten candidate MLs using the initial hyperparameter values for each ML method. Then, the three best performing MLs were chosen based on their performance among the ten ML models. The chosen three best performing MLs were further tuned automatically by altering their hyperparameters. A tire wear prediction algorithm was developed using the ensemble of the three best-performing models among the ten models (Figure 10a). Moreover, BPNN, which is not provided by AutoML, was trained separately to develop an algorithm to predict the amount of tire wear (Figure 10b and Table 4). The BPNN was constructed with a similar structure to that of Zhang et al. (2020) [4]. It is possible that only a subset of the features listed in Table 2 will be available depending on sensing configurations. Therefore, tire wear prediction algorithms were developed using various combinations of features in Figure 10 to compare the accuracies of the algorithm with respect to different combinations of information (Figure 10).

#### 3.4.3. Training and Testing of Wear Prediction Algorithm

All algorithms were trained and validated using 80% of the acceleration data set from the 180 simulations (Figure 5a). Then, the performance of the tire wear prediction algorithm was tested twice using the rest 20% of the acceleration data set from the 180 simulations (Figure 5a) and the acceleration data set from the 60 simulations (Figure 5b). The root mean square error (RMSE) was used as the metric for prediction errors (Equations (1) and (2)). The initial tread depth of the tire was 16 mm. For each wear prediction algorithm, learning and performance evaluation were repeated 60 times to calculate the average and standard deviation of the prediction error.

(1) $$Error=\sqrt{\sum_{i=1}^{N}\frac{{\left(wear_{prediction}-wear_{true}\right)}^{2}}{N}}$$

where

(2) $$wear=\frac{current\;tread\;depth-initial\;tread\;depth}{initial\;tread\;depth}$$

## 4. Results

### 4.1. Tire Model Validation

**Figure 11:**
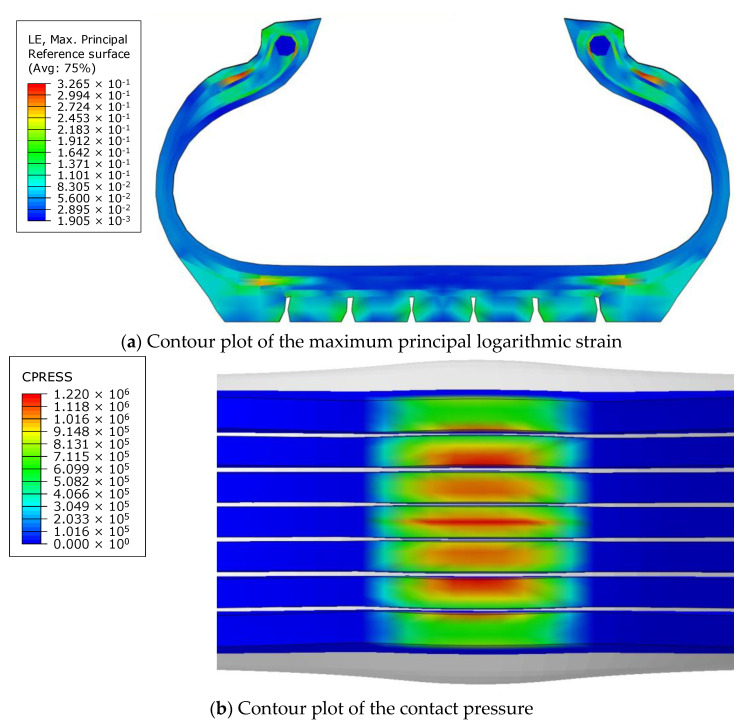
Tire simulation results for the vertical stiffness under the vertical load of 4150 kgf and internal pressure of 896 kPa.

**Figure 12:**
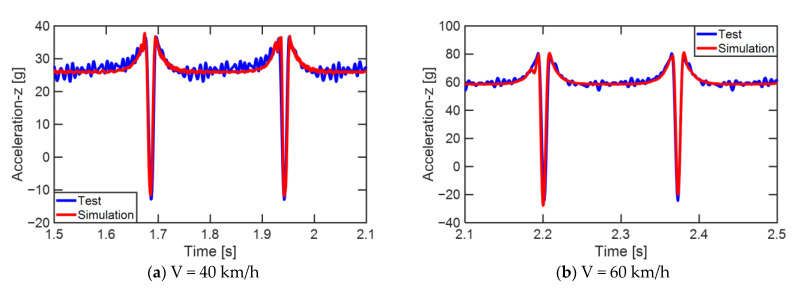
Comparison of the radial component of the internal tire accelerations under the drum rolling conditions.

The tire model exhibited vertical and lateral stiffnesses that were similar to those of the physical tire (Table 5). The tire model demonstrated typical maximum principal strain distributions with showing high numbers around bead turn-up edge areas and belt edge areas (Figure 11). The tire model demonstrated high contact pressure at the center region due to the large vertical load considering its size. The tire model resulted in behavior that was 4.1% and 5.4% stiffer than that of the physical tire under the vertical compression and lateral shear loading conditions, respectively. The tire model showed similar modal responses to those of the physical tire. The maximum error of the modal frequencies was 2.6% for the three internal pressure conditions (Table 6, Table 7 and Table 8). The modal shapes of the tire model matched well with those from the experiment up to three modes. The solid lines and contour plots in Table 6, Table 7 and Table 8 represent the modal shapes from the experiment and the simulations, respectively. Lastly, the tire model resulted in similar tire internal acceleration time histories with those from the physical tire under the drum rolling conditions with two different speeds (Figure 12).

### 4.2. Tire Wear Prediction

#### 4.2.1. Eight features from 1D-CNN

**Figure 13:**
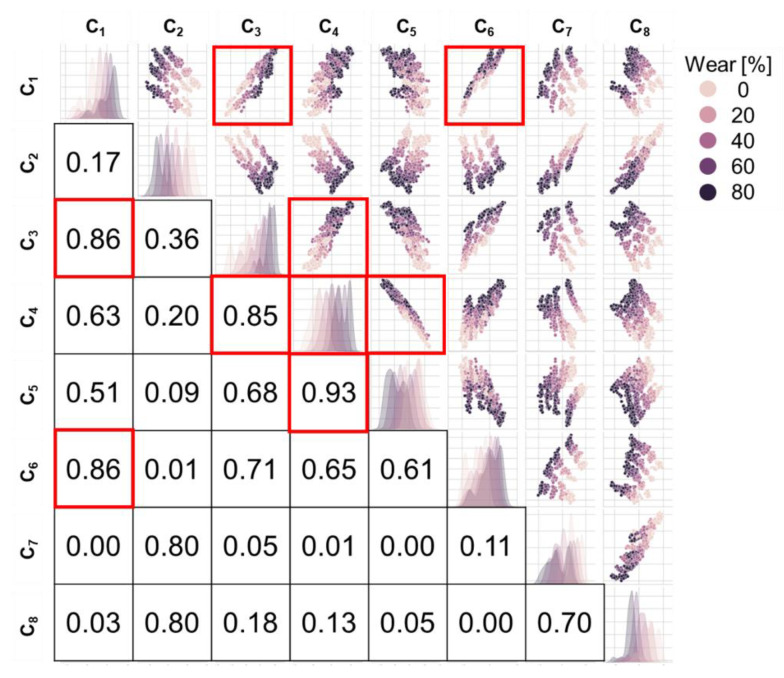
Pair plots between the eight features from 1D-CNN with wear amount. The pairs with coefficients of determination greater than 0.85 were indicated using squares.

Figure 13 shows pair plots of the eight features (C_1_ to C_8_) obtained from the 1D-CNN method with the wear amount indicated by the five different colors (Figure 8). The coefficients of determination (R^2^) between the pairs of features are also indicated in Figure 13, with pairs that have an R^2^ greater than 0.85 highlighted [29]. Clusters were formed based on the wear amount. For example, the pair plot between C_1_ and C_2_ or that between C_2_ and C_3_ clearly shows the clusters formed for each wear rate. The results showed that there were a few feature pairs, such as C_4_ and C_5_, with a correlation coefficient higher than 0.85 in terms of magnitude (Figure 13). It should be noted that including or excluding the correlated features did not affect the performance of the tire wear prediction algorithms.

#### 4.2.2. Prediction of Wear Amount of Tires

**Figure 14:**
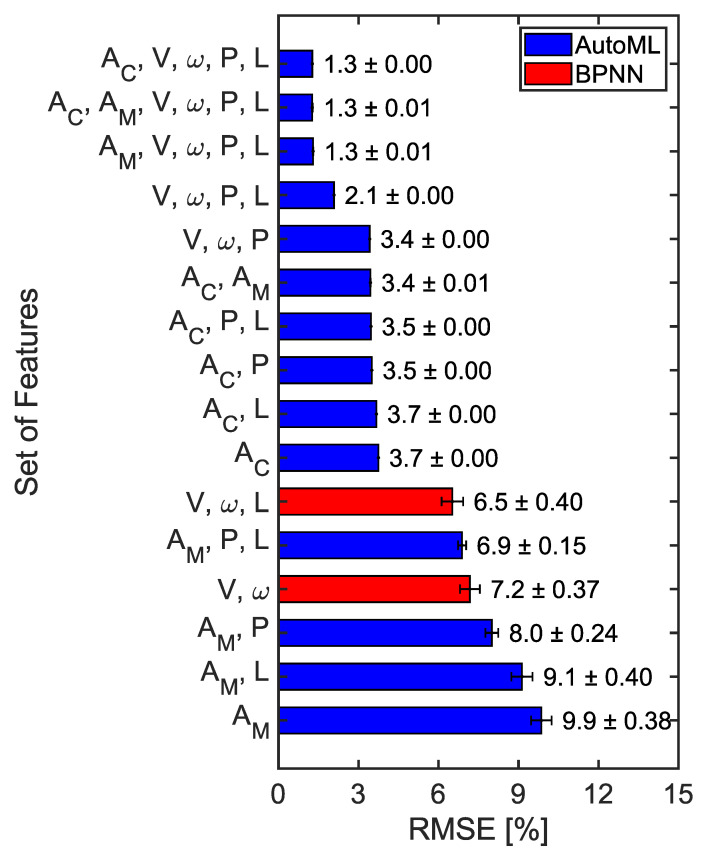
Comparison of the performance of the tire wear prediction with respect to the types of information. The numbers indicate average RMSE errors with plus and minus one standard deviation (A_C_: acceleration features extracted from the 1D-CNN, A_M_: manually selected acceleration features, V: wheel travel speed, ω: wheel rotational speed, P: tire internal pressure, L: tire vertical load).

**Figure 15:**
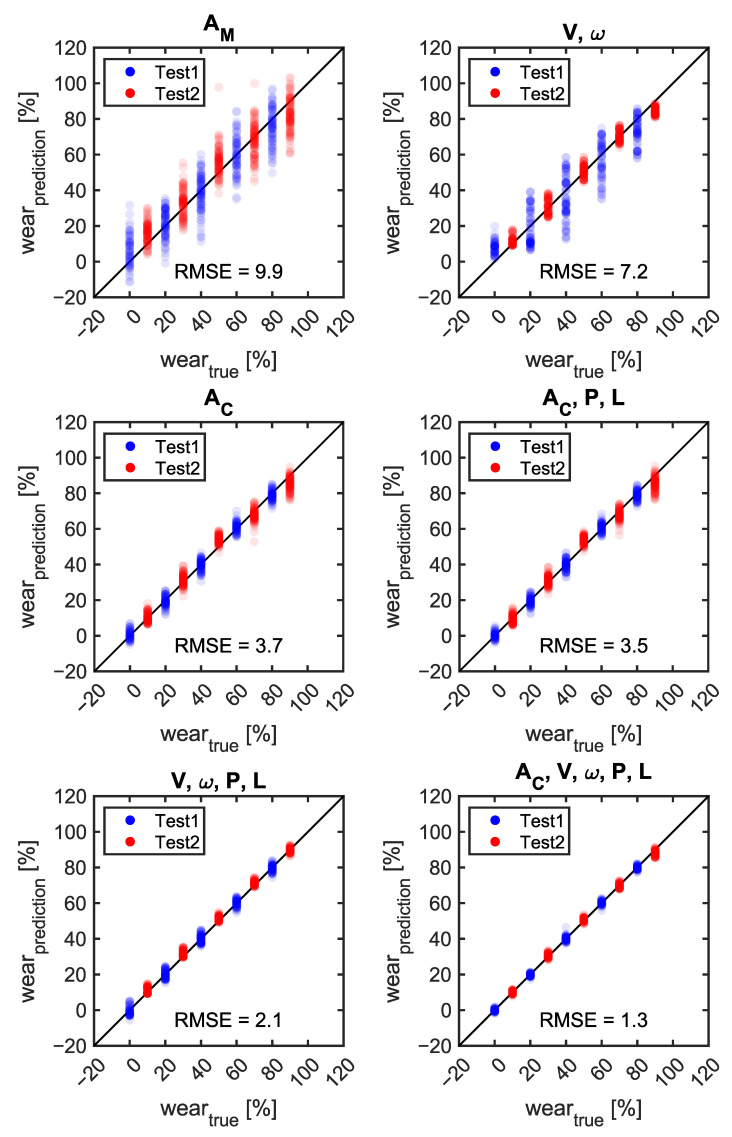
Scatter plots between the true and predicted wear amounts with respect to sets of features.

**Figure 16:**
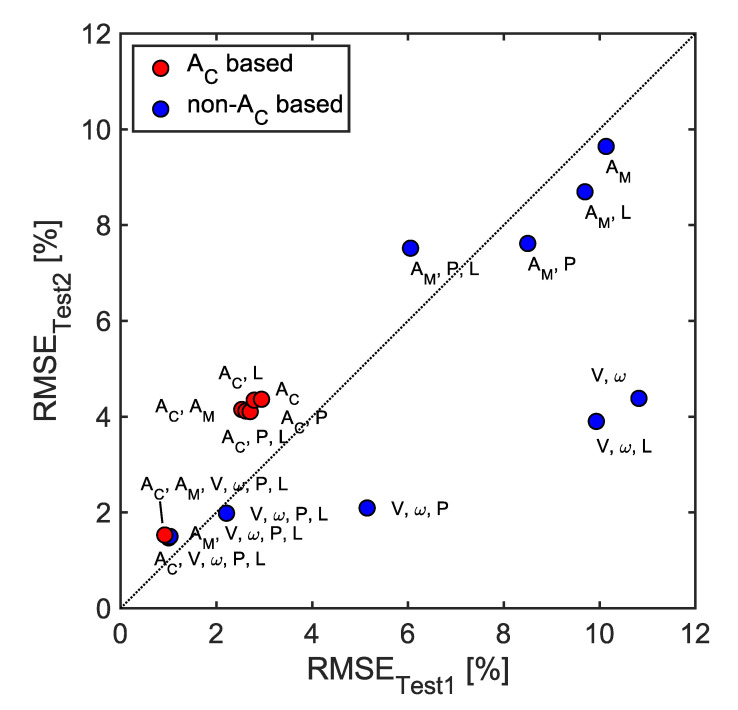
Comparison of prediction errors between the Test1 and Test2 data sets.

Figure 14 and Figure 15 compare the prediction error of the tire wear prediction algorithm according to the selection of the features. When using only wheel travel speed and wheel rotation speed, without any features related to the tire internal acceleration, the ensemble method was able to predict tire wear with an average RMSE error of 7.2% (1.2 mm) based on the 60 repetitions. When only the eight acceleration-related features (A_C_) which were extracted from the 1D-CNN were considered, the average RMSE error was 3.7% (0.60 mm). When only the manually selected acceleration features (A_M_) were considered, the average RMSE error was 9.9% (1.6 mm). The prediction accuracy improved as features related to tire internal pressure or tire vertical load were included. The tire wear prediction method based on the wheel travel and rotational speeds demonstrated the most improved performance when the internal pressure and vertical load features were added (2.1% or 0.34 mm). The lowest average RMSE error (1.3% or 0.21 mm) was obtained when the automatically selected acceleration features, wheel travel speed, wheel rotational speed, tire internal pressure, and tire vertical load were used to predict the tire wear amount. In general, the tire wear prediction algorithms developed using the data set from the first round of parametric tire rolling simulations demonstrated similar performance from Test1 and Test2, which suggests a good generalization performance (Figure 16). A_C_-based tire wear prediction algorithms tended to show higher RMSE for the Test2 data set than they did for the Test1 data set.

## 5. Discussion

The current study has four main differences from the previous tire wear prediction studies: consideration of various sensing options, the use of a 1D-CNN model with a bottleneck structure, the inclusion of an additional test, and the use of the finite element modeling approach [4,5,6,7]. 

### 5.1. Various Sensing Options

The current study not only developed algorithms for predicting tire wear amount but also quantitatively examined how their performance changes with the specific combination of information used (Figure 14). To the authors’ knowledge, there has been little study to investigate the benefit of the inclusion of a specific feature in predicting tire wear. Previous studies presented promising results using a pre-selected set of features in predicting tire wear state based on either effective rolling radius or tire-mounted sensor data. It was difficult to compare the expected performance of various approaches because of the different test conditions and algorithms used in each study. The comparison results can guide researchers in selecting optimal sensing options depending on their circumstances. V and ω-based methods demonstrated relatively good performance compared to acceleration-based methods (Figure 14). When only information on the travel speed (V) and the wheel rotation speed (ω) were used the average RMSE error was 7.2% (1.15 mm). The wheel travel speed and the wheel rotational speed can be obtained from the GPS (Global Positioning System) and IMU (Inertial Measurement Unit) and wheel speed sensor, respectively. Ding and Wang (2011) showed that the velocity of a vehicle can be estimated with velocity standard deviation errors of 2.9–6.7 mms^−1^ [30]. When tire internal pressure (P) was added to the feature set, the average RMSE was reduced to 3.4% (0.54 mm) from 7.2%. When V, ω, P, and L were used as the feature set, the accuracy of the tire wear prediction algorithm was 2.1% (0.34 mm). This finding is promising because these three features can be obtained from the sensors that are already available in recent vehicles. Wear prediction based on the wheel travel speed and wheel rotational speed is based on the estimation of the effective rolling radius of a tire. That is why the performance of the V and ω-based method was sensitive to the inclusion of the tire internal pressure or the tire vertical load. Sabatini et al. showed that the rolling radius of a motorcycle tire can be estimated using motorcycle speed from GPS, tire pressure, and wheel rotational speed for traction control purposes rather than the estimation of the tire wear amount [31]. Poloni and Lu (2017) also showed that the effective rolling radius can be predicted based on ω and V, but there was a limitation in that this method was only examined for a new tire, i.e., for one tread depth condition [7]. In practice, the wheel torques applied by either the powertrain or brake system need to be considered to compensate for the longitudinal slip of the tire.

When only tire acceleration-related features were considered, tire wear prediction based on A_C_ exhibited higher accuracy (3.7%) than those of the A_M_ (9.9%) and V and ω features (7.2%) (Figure 14). These results indicate that the A_C_ only method can be a good choice to predict the amount of tire wear when other features such as V, ω, P, or L are unavailable. The accuracy of the A_M_-based method of the current study was inferior to previous studies although similar features with previous studies were considered in the current study. Zhang et al. were able to predict the amount of tire wear within less than a prediction error of 1% using manually selected features from the tire internal acceleration and strain data [4]. The large discrepancy between the performances of the current study and Zhang et al. may stem from the lack of consideration of the tire strain data in the current study. Therefore, it would be worthwhile to investigate the effect of the strain gauge data in predicting tire wear if the strain gauge is a viable option [32]. It should be also noted that adding more sensors in the tire increases the chance of failure, power consumption, and cost of the tire monitoring system.

As more features were considered, the accuracies of tire wear prediction algorithms were improved (Figure 14). The performance of the A_M_-based method with the P and L showed improved accuracy from 9.9% to 6.9%. The performance of the V and ω-based method with the P and L showed improvement in the prediction accuracy from 7.2% to 2.1%. When all of the vehicle and tire information (Table 2) are used, the combination of either A_C_, V, ω, P, and L or A_M_, V, ω, P, and L demonstrated the highest accuracy, with an average wear prediction error of 1.3% (0.21 mm). However, it should be noted that, even by using V, ω, P, and L, the wear prediction error was 2.1% (0.34 mm), showing similar performance to that of the A_C_, V, ω, P, and L feature sets. Therefore, the V, ω, P, and L-based tire wear prediction methods, which can be implemented using vehicle-mounted sensors and TPMS, seemed to be the most efficient way among the methods considered in the current study. It should be also noted that the tire internal acceleration signals can be used to estimate other useful information such as the cornering stiffness of the tire, tire vertical load, and the friction coefficient between the tire and the road, etc. [33,34]. In general, the use of automated machine learning (AutoML) resulted in a smaller RMSE than the backpropagation neural network (BPNN). This result suggests that it is worthwhile to try various machine learning algorithms.

### 5.2. 1D-CNN with Bottleneck Structure

Acceleration-related features for predicting tire wear were automatically extracted using the 1D-CNN with a bottleneck structure. It may be difficult to ensure that the manually selected features of the acceleration traces based on previous knowledge would contain all the important information of the acceleration signals for predicting tire wear (Table 2 and Figure 8). The manually selected acceleration features did not capture the subtle curvatures of the acceleration signals, particularly for the a_z_ component (Figure 7). The relatively poor wear prediction performance based on A_M_ (RMSE = 9.9%) indicates that A_M_ contains insufficient information to predict tire wear amount. An attempt was made to extract characteristics of the tire internal acceleration signals using 1D-CNN without subjective decisions such as selecting maxima or minima. Huang et al. utilized 1D-CNN to classify textile fibers [35]. Sateesh Babu et al. demonstrated that CNN can be used to estimated remaining useful life [36]. Tire wear prediction based on A_C_ exhibited higher accuracy (3.7%) than those of the A_M_ (9.9%) (Figure 14 and Table 2). The good performance of the tire wear prediction based on the A_C_ features was anticipated in Figure 13. Clear clusters of different wear amounts were observed in a couple of pair plots such as between C_1_ and C_2_, C_2_ and C_3_, and C_2_ and C_5_. These results imply that the A_C_, which is a set of the acceleration-related bottleneck features extracted by the 1D-CNN, contains more information on the amount of tire wear than the A_M_. Additionally, CNN with a bottleneck structure is often used to reduce computation time, reduce noise, and so on [37]. In the current study, a bottleneck layer was used to merge the high-level abstract representation of the acceleration signal (A_C_) with other information, such as travel speed, tire internal pressure and tire vertical load, to predict tire wear amounts [38]. As a result, tire wear prediction using A_C_, V, ω, P, and L demonstrated the best performance (RMSE = 1.3%). To the authors’ knowledge, there has been no study to apply 1D-CNN with a bottleneck structure in predicting tire wear.

### 5.3. Generalization Performance

The current study evaluated the ability of the developed tire wear prediction algorithms to generalize to new data in two steps, called Test1 and Test2 (Figure 16). To assess the generalization performance of the tire wear algorithms, a second test was conducted using a data set (Test2) that was distinct from the training and validation data sets in terms of tire wear amounts (Table 1 and Figure 5b). In contrast, previous studies used data from the tires with the same sets of tire wear amounts for training, validation, and test [4,5,6]. For example, if ten acceleration cycles were obtained from a single tire, six of them might be used for training, two for validation, and the remaining two for testing. These three divided data sets might not be diverse enough to effectively test a developed tire wear prediction algorithm, potentially impacting the accuracy of the results. In general, the tire wear prediction algorithms developed using the data set from the first round of parametric tire rolling simulations demonstrated a good generalization performance (Figure 16). However, A_C_-based methods showed a slight overfitting tendency while the non-A_C_-based methods did not (Figure 16). Therefore, it can be concluded that the current data query method, which utilizes train, validation, and test sets from the same sets of tires, is valid for the tire wear prediction problem.

### 5.4. FE Tire Model for Intelligent Tire Technology

The current study demonstrated that a validated FE tire model can simulate the tire internal accelerations from a tire rolling experiment (see Section 4.1). The reliability of the tire internal acceleration data was ensured by validating the tire model under static and dynamic test results. Most importantly, the tire model demonstrated tire internal acceleration that was similar to those of the physical tire under the drum rolling conditions with speeds of 40 km/h and 60 km/h (Figure 7). The extensively validated tire model was used for generating tire internal acceleration signals under various driving, loading, and tire wear conditions. In fact, this FE tire model did not go through extensive validation processes with multiple iterations after it was initially modeled based on the CAD data and material property information. The validation results indicated that a concept of smart tire technology based on tire internal acceleration could be investigated using FE analyses. This fact will be particularly useful for heavy-duty tires, which are more difficult to conduct experiments on than passenger tires due to the required load capacity. Braghin et al. determined the locations of accelerometers inside of the tire using a validated FE tire model to estimate contact forces and contact patch features [10].

### 5.5. Limitations

The conclusions of the current study were based on the simulation results under simple straight driving conditions. In the current study, the wear pattern of the tire is assumed to be perfectly uniform (Figure 5). Additionally, the tire was perfectly upright during the tire rolling simulations with zero camber angle. However, in practice, tires do not wear out perfectly uniformly and run with some camber angles. Additionally, the performance of the tire wear prediction algorithm was evaluated without considering the sensing error. The impacts of these limitations need to be evaluated by conducting experimental studies in the future.

## 6. Conclusions

In the current study, the performances of tire wear prediction algorithms were quantitatively compared based on different combinations of sensing information from the vehicle and tire. As expected, the accuracy of the tire wear prediction was found to be highest when vehicle and tire information were all aggregated together. It was found that the accuracy of tire wear prediction was highest when all vehicle and tire information was used together. Interestingly, almost similar accuracy was obtained using only wheel travel speed, wheel rotation speed, tire pressure, and tire vertical load information, which can be obtained without additional sensors in current vehicles. Additionally, acceleration-related features extracted from 1D-CNN with a bottleneck structure seemed to be a good choice when tire internal pressure or tire vertical load was unavailable. The tire driving data used to develop the tire wear prediction algorithm was obtained from a finite element tire model, which was shown to predict tire internal acceleration in a manner similar to actual tire performance. This result indicates that the finite element tire model can be effectively utilized to investigate future intelligent tire technology concepts. Lastly, the conclusion of this study needs to be verified through experimental studies. 

## Figures and Tables

**Table 1:** Simulation parameters for training machine learning algorithms for tire wear prediction.

Purpose	Parameter	Values	Number of Simulations
Training, Validation, and Test1	Tread wear [%] (100% = 16 mm)	0, 20, 40, 60, 80	180
	Velocity [km/h]	40, 60, 80, 100	
	Pressure [kPa]	717, 896, 1076	
	Load [kgf]	2400, 3000, 3600	
Test2	Tread wear [%] (100% = 16 mm)	10, 30, 50, 70, 90	60
	Velocity [km/h]	40, 60, 80	
	Pressure [kPa]	806, 896	
	Load [kgf]	2700, 3000	

**Table 2:** List of features for machine learning-based wear prediction algorithm.

Potential Source	Name		Description
Vehicle	Wheel travel speed (V)		Travel speed at wheel center
	Wheel rotational speed (ω)		Wheel rotational speed
	Vertical force (L)		Normal force at the contact patch
TPMS	Tire pressure (P)		Tire internal pressure
Tire internal accelerometer	A_M_	a_x_A	Circumferential acceleration (a_x_) value at the beginning of the cycle
		a_x_B_t_	Onset time for the first rise of the a_x_ in the current cycle
		a_x_B	a_x_ value at axB_t_
		a_x_C_t_	Time for the first local maximum a_x_ value in the current cycle
		a_x_C	First local maximum a_x_ value in the current cycle
		a_x_D_t_	Time for the first local minimum a_x_ value in the current cycle
		a_x_D	First local minimum a_x_ value in the current cycle
		a_x_E_t_	Time for the second local maximum a_x_ value in the current cycle
		a_x_E	Second local maximum a_x_ value in the current cycle
		a_x_dt_BC_	Time period between the a_x_A and a_x_B
		a_x_dt_CD_	Time period between the a_x_C and a_x_D
		a_x_dt_DE_	Time period between the a_x_D and a_x_E
		a_x_dy_BC_	Difference between a_x_B and a_x_C
		a_x_dy_CD_	Difference between a_x_C and a_x_D
		a_x_dy_DE_	Difference between a_x_D and a_x_E
		a_x_sl_BC_	Rate of change between points a_x_B and a_x_C
		a_x_sl_CD_	Rate of change between points a_x_C and a_x_D
		a_x_sl_DE_	Rate of change between points a_x_D and a_x_E
		a_z_A	Radial acceleration (a_z_) value at the beginning of a cycle
		a_z_Bt	Time for the first local maximum a_z_ in the current cycle
		a_z_B	First local maximum a_z_ in the current cycle
		a_z_Ct	Time for the minimum a_z_ in the current cycle
		a_z_C	Minimum a_z_ in the current cycle
		a_z_Dt	Time for the second local maximum a_z_ in the current cycle
		a_z_D	Second local maximum a_z_ in the current cycle
		a_z_dt_BD_	Time period between the a_z_B and a_z_D
		a_z_dt_BC_	Time period between the a_z_B and a_z_C
		a_z_dt_CD_	Time period between the a_z_C and a_z_D
		a_z_dt_DE_	Time period between a_z_D and a_z_E
		a_z_dy_BC_	Difference between a_z_B and a_z_C
		a_z_dy_CD_	Difference between a_z_C and a_z_D
		a_z_dy_DE_	Difference between a_z_D and a_z_E
		a_z_sl_BC_	Rate of change between points a_z_B and a_z_C
		a_z_sl_CD_	Rate of change between points a_z_C and a_z_D
		a_z_sl_DE_	Rate of change between points a_z_D and a_z_E
	A_C_	C_1_~C_8_	Eight features generated from 1D-CNN

**Table 3:** List of altered parameters from the default values for AutoML method (PyCaret 2.3.10).

Step	ID	Description	Value
Setup	1	train_size	0.8
	2	polynomial_features	True
	3	feature_interaction	True
	4	remove_multicollinearity	True
	5	multicollinearity_threshold	0.975
	6	fold	10
Compare_models	1	sort	‘RMSE’
	2	N_select	3
	3	include	[“lr”, “lar”, “huber”, “ada”, “omp”, “ridge”, “lasso”, “llar”, “br”, “en”, “par”]
Tune_model	1	optimize	‘RMSE’
Blend_models	1	fold	10
	2	optimize	‘RMSE’

**Table 4:** Hyper parameters of the BPNN for tire wear prediction.

ID	Description	Value
1	Optimizer	adam
2	Learning rate	0.001
3	Loss	MSE
4	epochs	5000
5	batch_size	1024
6	restore_best_weights	True

**Table 5:** Comparison of stiffness of tires from the experiment and the simulation.

	Vertical			Lateral		
	Displacement at 4150 kgf (mm)	Stiffness (kgf/mm)	Error (%)	Displacement (mm)	Stiffness (kgf/mm)	Error (%)
Test	42.8	97	-	11	37	-
FEM	41.1	101	4.1	11	39	5.4

**Table 6:** Comparison of the modal frequencies and modal shapes between the experiment and simulation (Pressure 85%).

Pressure	1st Mode		2nd Mode		3rd Mode	
85%	Test	Simulation	Test	Simulation	Test	Simulation
Frequency (Hz)	77	75	99	98	121	121
Error (%)	−2.6		−1.0		0.0	
Mode Shape	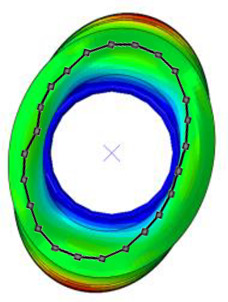		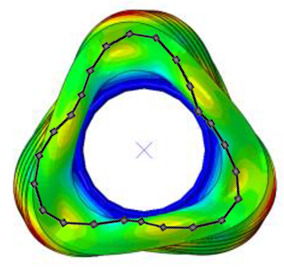		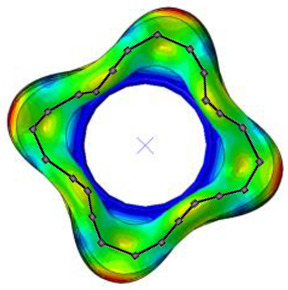	

**Table 7:** Comparison of the modal frequencies and modal shape between the experiment and simulation (Pressure 100%).

Pressure	1st Mode		2nd Mode		3rd Mode	
100%	Test	Simulation	Test	Simulation	Test	Simulation
Frequency (Hz)	82	80	105	104	130	130
Error (%)	−2.5		−1.0		0.0	
Mode Shape	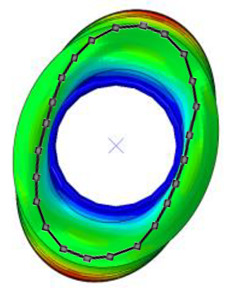		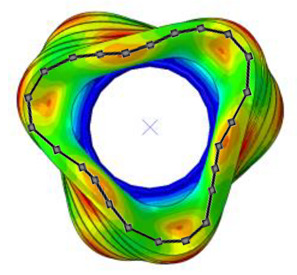		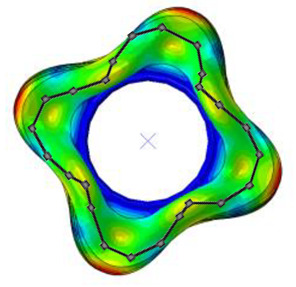	

**Table 8:** Comparison of the modal frequencies and modal shape between the experiment and simulation (Pressure 115%).

Pressure	1st Mode		2nd Mode		3rd Mode	
115%	Test	Simulation	Test	Simulation	Test	Simulation
Frequency (Hz)	85	85	110	112	136	138
Error (%)	0.0		1.8		1.5	
Mode Shape	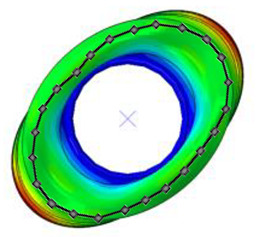		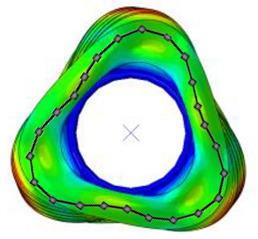		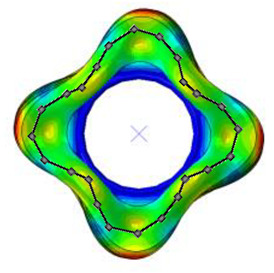	

## Data Availability

Not applicable.

## Appendix A

Table A1 shows the list of default parameters for the ten ML methods provided by the AutoML function in PyCaret (version 2.3.10) [19]. Table A2 shows the list of default parameters for the AutoML method itself rather than specific ML method.

**Table A1:** Default Values of Hyper Parameters Used for the AutoML method (PyCaret version 2.3.10) [16].

ID	Machine Learning Method	Default Values of Hyper Parameters
1	Linear Regression	fit_intercept = True, n_jobs = −1, normalize = False
2	Lasso Regression	alpha = 1.0, fit_intercept = True, max_iter = 1000, normalize = False, positive = False, precompute = False, random_state = 1, selection = ‘cyclic’, tol = 0.0001, warm_start = False
3	Ridge Regression	alpha = 1.0, fit_intercept = True, max_iter = None, normalize = False, random_state = 1, solver = ‘auto’, tol = 0.001
4	Elastic Net	alpha = 1.0, fit_intercept = True, l1_ratio = 0.5, max_iter = 1000, normalize = False, positive = False, precompute = False, random_state = 1, selection = ‘cyclic’, tol = 0.0001, warm_start = False
5	Lasso Least Angle Regression	alpha = 1.0, eps = 2.220446049250313 × 10^−16^, fit_intercept = True, fit_path = True, jitter = None, max_iter = 500, normalize = True, positive = False, precompute = ‘auto’, random_state = 1, verbose = False
6	Orthogonal Matching Pursuit	fit_intercept = True, n_nonzero_coefs = None, normalize = True, precompute = ‘auto’, tol = None
7	Bayesian Ridge	alpha_1 = 1 × 10^−6^, alpha_2 = 1 × 10^−6^, alpha_init = None, compute_score = False, fit_intercept = True, lambda_1 = 1 × 10^−6^, lambda_2 = 1 × 10^−6^, lambda_init = None, n_iter = 300, normalize = False, tol = 0.001, verbose = False
8	Passive Aggressive Regressor	C = 1.0, average = False, early_stopping = False, epsilon = 0.1, fit_intercept = True, loss = ‘epsilon_insensitive’, max_iter = 1000, n_iter_no_change = 5, random_state = 1, shuffle = True, tol = 0.001, validation_fraction = 0.1, verbose = 0, warm_start = False
9	Huber Regressor	alpha = 0.0001, epsilon = 1.35, fit_intercept = True, max_iter = 100, tol = 1 × 10^−5^, warm_start = False
10	AdaBoost Regressor	base_estimator = None, learning_rate = 1.0, loss = ‘linear’, n_estimators = 50, random_state = 1

**Table A2:** Parameters for Automated Machine Learning (PyCaret version 2.3.10).

ID	Description	Value
1	Session_id	1
2	Target	wear
3	Original Data	(1485, 723)
4	Missing Values	FALSE
5	Numeric Features	722
6	Categorical Features	0
7	Ordinal Features	FALSE
8	High Cardinality Features	FALSE
9	High Cardinality Method	None
10	Transformed Train Set	(1188, 27)
11	Transformed Test Set	(297, 27)
12	Shuffle Train-Test	TRUE
13	Stratify Train-Test	FALSE
14	Fold Generator	KFold
15	Fold Number	10
16	CPU Jobs	−1
17	Use GPU	TRUE
18	Log Experiment	FALSE
19	Experiment Name	reg-default-name
20	USI	3746
21	Imputation Type	simple
22	Iterative Imputation Iteration	None
23	Numeric Imputer	mean
24	Iterative Imputation Numeric Model	None
25	Categorical Imputer	constant
26	Iterative Imputation Categorical Model	None
27	Unknown Categoricals Handling	least_frequent
28	Normalize	FALSE
29	Normalize Method	None
30	Transformation	FALSE
31	Transformation Method	None
32	PCA	FALSE
33	PCA Method	None
34	PCA Components	None
35	Ignore Low Variance	FALSE
36	Combine Rare Levels	FALSE
37	Rare Level Threshold	None
38	Numeric Binning	FALSE
39	Remove Outliers	FALSE
40	Outliers Threshold	None
41	Remove Multicollinearity	TRUE
42	Multicollinearity Threshold	0.975
43	Remove Perfect Collinearity	TRUE
44	Clustering	FALSE
45	Clustering Iteration	None
46	Polynomial Features	FALSE
47	Polynomial Degree	None
48	Trignometry Features	FALSE
49	Polynomial Threshold	None
50	Group Features	FALSE
51	Feature Selection	FALSE
52	Feature Selection Method	classic
53	Features Selection Threshold	None
54	Feature Interaction	FALSE
55	Feature Ratio	FALSE
56	Interaction Threshold	None
57	Transform Target	FALSE
58	Transform Target Method	box-cox
